# State Registration in Great Britain

**Published:** 1916-05-27

**Authors:** 


### THE MATRONS' AND SISTERS' DEPARTMENT.

### STATE REGISTRATION IN GREAT BRITAIN.

### THE CONFERENCE AND ITS OUTLOOK.

In the afternoon of the 19th inst., at the Automobile Club, Pall Mall, the first meeting of the Conference arranged between representatives of the College of Nursing and those of the Society for the State Registration of Trained Nurses was held. The nine representatives of the College of Nursing were:—Miss Annie McIntosh, matron of St. Bartholomew's Hospital; Miss Lloyd Still, matron of St. Thomas's Hospital and the Nightingale Training School for Nurses; Miss Haughton, matron of Guy's Hospital; Miss Cox-Davies, matron of Royal Free Hospital; Miss Barton, Poor-Law Nurses; Miss Amy Hughes, general superintendent Queen Victoria's Jubilee Institute for Nurses; Sir E. Cooper Perry, M.D., F.R.C.P., Mr. Comyns Berkeley, M.D., F.R.C.P., M.C. (Royal British Nurses' Association), and Dr. Turney, M.D., F.R.C.P., F.R.C.S. The representatives of the Society for State Registration of Nurses were:—Mrs. Bedford Fenwick, Miss M. Heather Bigg, Matron's Council; Miss M. Breay, Miss L. A. Morgan, Fever Nurses' Association; Mrs. Porter, Irish Nurses' Association; Major Chapple, M.P.; Dr. J. Macgregor Robertson, Scottish Nurses' Association; Dr. Bezly Thorne, Royal British Nurses' Association; and Mr. T. Jenner Verrall, British Medical Association.

It may be noted, in this connection, that Miss Beatrice Kent, formerly night sister of the Guest Hospital, Dudley, which has an average of seventy-six occupied beds, who is a member of the State Registration Society, gave an *ex parte* address on the 12th inst., containing several statements which have been corrected more than once, to nurses and others assembled at the York County Hospital. It should be clear to the Council of the State Registration of Nurses' Society that meetings of this kind should not be held, or addresses of the type in question made under its auspices, at all, or at least during the sittings of the Conference on State Registration now being held, its object being, if possible, to secure the united action of all sections of the nursing profession on this question.

It was, we understand, agreed that the discussions at the meeting of the Conference on the 19th inst. should not be published at present. We therefore hold over our report, in accordance with what we are informed was the general wish of those present.

